# Cost items in melanoma patients by clinical characteristics and time from diagnosis

**DOI:** 10.3389/fonc.2023.1234931

**Published:** 2023-11-08

**Authors:** Alessandra Buja, Claudia Cozzolino, Anna Zanovello, Ruggero Geppini, Andrea Miatton, Manuel Zorzi, Mariagiovanna Manfredi, Emanuela Bovo, Paolo Del Fiore, Saveria Tropea, Luigi dall’Olmo, Carlo Riccardo Rossi, Simone Mocellin, Marco Rastrelli, Massimo Rugge

**Affiliations:** ^1^ Department of Cardiologic, Vascular and Thoracic Sciences, and Public Health, University of Padua, Padua, Italy; ^2^ Soft-Tissue, Peritoneum and Melanoma Surgical Oncology Unit, Veneto Institute of Oncology (IOV), Istituto di Ricovero e Cura a Carattere Scientifico (IRCCS), Padua, Italy; ^3^ Veneto Tumor Registry, Azienda Zero, Padua, Italy; ^4^ Department of Surgery, Oncology and Gastroenterology (DISCOG), University of Padua, Padua, Italy; ^5^ Pathology and Cytopathology Unit, Department of Medicine-DIMED, University of Padua, Padua, Italy

**Keywords:** melanoma, direct costs, specific costs, cost of illness, health economics, healthcare assessment, healthcare services research

## Abstract

**Background:**

Costs related to the care of melanoma patients have been rising over the past few years due to increased disease incidence as well as the introduction of innovative treatments. The aim of this study is to analyse CMM cost items based on stage at diagnosis, together with other diagnostic and prognostic characteristics of the melanoma.

**Methods:**

Analyses were performed on 2,647 incident cases of invasive CMM that were registered in 2015 and 2017 in the Veneto Cancer Registry (RTV). Direct melanoma-related costs per patient were calculated for each year ranging from 2 years before diagnosis to 4 years after, and were stratified by cost items such as outpatient services, inpatient drug prescriptions, hospital admissions, hospice admissions, and emergency room treatment. Average yearly costs per patient were compared according to available clinical-pathological characteristics. Lastly, log-linear multivariable analysis was performed to investigate potential cost drivers among these clinical-pathological characteristics.

**Findings:**

Overall, the average direct costs related to melanoma are highest in the first year after diagnosis (€2,903) and then decrease over time. Hospitalization costs are 8 to 16 times higher in the first year than in subsequent years, while the costs of outpatient services and inpatient drugs decrease gradually over time. When stratified by stage it is observed that the higher expenditure associated with more advanced stages of CMM is mainly due to inpatient drug use.

**Conclusion:**

The results of the present study show that grouping patients according to tumour characteristics can improve our understanding of the different cost items associated with cutaneous malignant melanoma. CMM patients experience higher costs in the first year after diagnosis due to higher hospitalization and outpatient services. Policy makers should consider overall and stage-specific annual costs when allocating resources for the management of CMM patients.

## 1 Introduction

Cutaneous malignant melanoma (CMM) is among some of the most common cancers in young adults (age 20-39) (1–3). Even though mortality due to cutaneous melanoma has been significantly decreasing over the past decade, its incidence has continued to rise at a steady pace (4). In the United States, the rate of new cases has more than tripled since 1975, and in 2021 cutaneous melanoma accounted for 5.6% of all new cancer cases (5, 6).This increase in overall CMM rate, as well as the introduction of innovative therapies for its treatment, means that CCM is a rising public health issue with a significant financial and social burden (7–9). However, health care pathways for CMM patients vary widely depending on their cancer stage (7, 10). While most thin melanomas can often be cured with simple wide excision, more advanced forms require complex treatments, implying quite a different burden of health care resource consumption (11, 12). In an effort to ensure that all patients receive the most effective therapies, policy-makers need to address the issue of sustainability of our healthcare systems as a whole (13–15).

There are many recent studies focusing on direct CMM costs, but few of them have analysed the differences in real-world costs associated with a comprehensive health care pathway covering all the stages of the disease (6, 14–17). Furthermore, very few studies have been conducted in Europe on the stage-specific direct health care costs of CMM (some that have done so include Lyth et al. and Buja et al.) (15–17).

This study aims to analyse the real-world direct costs related to a population-based CMM cohort of patients. Analyses will involve both overall costs as well as item-specific costs, stratifying the analyses by the cancer stage at diagnosis, patient survival, as well as other tumour characteristics, with a four-year follow-up after diagnosis.

## 2 Methods

### 2.1 Context

The Italian public healthcare system is managed regionally, providing universal coverage at the point of delivery, which is largely free of charge and primarily supported by general taxation (18). Its policies are grounded on the fundamental values of universality, free access, freedom of choice, pluralism in provision, and equity.

Veneto is an Italian region located in the North-eastern area of the country, with a population of 4.9 million residents and a mean age of 54.4 years. Considering the latest available socio-economic indicators, the Veneto region has a per capita gross domestic product (GDP) of 33,800 euros, with an unemployment rate of 5.8%, and 29.3% of Veneto inhabitants aged 30-34 have a university degree (11, 19).

In 2015, the Veneto Oncology Network (ROV) detailed the complete procedures for the clinical management of CMM patients in a document based on the current national and international literature (20–23). It defines standardized clinical care pathways from diagnosis to end-of-life care, as well as a set of indicators to assess the quality of care by evaluating the consistency between recommendations and real-world clinical practice (24).

### 2.2 Clinical and cost data

This population-based cohort study includes all 2015 and 2017 incident cases of cutaneous malignant melanoma recorded by the Veneto Cancer Registry (RTV) in the cutaneous malignant high-resolution regional cancer registry, which provides demographic coverage of the entire region on a two-year basis.

The RTV recording procedures rely on various informative sources such as pathology reports, clinical charts, death certificates, and health service administrative records. The following variables were extracted by high resolution registry for the present study: age, sex, 8th edition AJCC TNM stage at diagnosis, and CMM histological subtypes.

The cost analysis was conducted from a health system perspective. Data on visits to outpatient clinics, specialist services, drug prescriptions, hospital or hospice admissions, treatments at the emergency department, and the use of medical devices were obtained from the regional administrative subject-level databases (see below). The cost of any diagnostic or therapeutic (surgical or other) interventions was based on the reimbursement rates established by the Veneto Regional Authority. For cost assessment we considered the following sources:

- The Outpatient database, which contains the information on all medical procedures (i.e., specialist visits, laboratory and radiological tests, radiotherapy sessions, etc.), excluding inpatient care, delivered at outpatient facilities under NHS funding. These procedures are valued at the rate stated in the Tariff Nomenclature for outpatient services (TNOS), which is a detailed formulary of medical procedures for outpatients. These services are categorised within the cost item labelled as “Outpatient services”.
- The Hospital Admissions database, which includes the diagnosis-related group (DRG) for each admission, valued at the rate indicated in the Tariff Nomenclature for inpatient services (TNIP), which is a formulary covering all hospital activities, including day hospital admissions, preliminary pre-operative exams, and sentinel lymph node staging procedures. All services provided during hospitalization, whether in a day hospital or inpatient setting, are categorised under the cost item “Hospitalization”.
- Regional databases of outpatient drug prescriptions and in-hospital drug consumption, which record the costs of all medical therapies, encompassing dosage details. Also included are “high-cost” chemotherapeutic agents (i.e., interferon, pembrolizumab, nivolumab, vemurafenib, Osimertinib, dabrafenib, trametinib, ipilimumab, binimetinib, temozolomide, fotemustine, paclitaxel, melphalan) and all associated supportive therapies. The cost item associated with them is referred to as “Inpatient drugs”.
- The Emergency Department Admissions database, which records the cost of each admission as the sum of all medical procedures undertaken. The data generated is allocated to the cost item classified as “Emergency department visit”.
- The Hospice database, which records the length of stay. The corresponding cost item is classified as “Hospice”.

These distinct databases and their cost categorizations contribute to a more comprehensive analysis of healthcare expenditure patterns in the studied context, which is the Italian healthcare system.

Each patient was linked via a unique and anonymous identification code to all administrative data (see the cost assessment sources list above). All costs sustained from two years before melanoma diagnosis up to four years after diagnosis were included. The average real-world costs per year per patient (total and per single item of expenditure) were calculated and stratified by the following clinical variables: site of primary tumour, histological phenotype, and TNM stage at initial cancer assessment.

### 2.3 Statistical analysis

Differences in total costs, item expense distribution (comparing < 64 vs. 65-year-old patients), TNM stage, CMM histology subtype (i.e., nodular versus non-nodular), and survival time were also assessed by stratifying cost data by these variables.

Costs are summarized as survival-weighted means and medians, 95% confidence intervals for mean and minimum–maximum intervals are also provided.

Survival-weighted costs were calculated (for each year and for the entire period observed) by dividing the sum of total incurred costs by the observed person-time, i.e., the sum of total time (days of the year) in which each patient was alive, and then transformed to person-years.

A log-linear multivariable analysis was performed to investigate potential cost drivers among the available clinic-pathological characteristics. The mean person-years melanoma-specific costs sustained from diagnosis to 4 years after diagnosis defined the output variables of four different models adapted to the data. Costs were transformed to the logarithmic in order to manage the skewed distribution and meet linear regression assumptions. The independent variables considered were age, sex, TNM stage at diagnosis, mitotic count, ulceration, regression, growth phase, TILs, together with tumour location, histology subtype, and time to death.

Results were deemed statistically significant at the p < 0.05 level. Data preparation, analyses and visualizations were conducted using R (25) and Python (26).

## 3 Results

A total of 2,647 cutaneous malignant melanoma incident cases were included in this study, of which 1,279 cases were diagnosed in 2015 (48.3%) and 1,368 in 2017 (51.7%). The mean age at diagnosis was 59.7 (SD ± 16.2) years. The overall survival at 4 years was 86.32%. Less than 15% of the patients presented with an advanced TNM stage at diagnosis (9.9% in stage III, 3.5% in stage IV). The sample characteristics are summarized in **Table 1**.

**Table 1:** Descriptive sample statistics.

	Value	% (N = 2,647)
Age group		
<18	5	0.19
18-64	1,564	59.09
≥65	1,078	40.73
Sex		
Male	1,404	53.0
Female	1,243	47.0
Primary site		
Trunk	1,273	48.1
Lower limb	508	19.2
Upper limb	367	13.9
Head	284	10.7
Hands/feet	119	4.5
Unknown	96	3.6
M. Histology Subtype		
Superficial spreading	1,876	70.9
Nodular	365	13.8
Malignant	228	8.6
Lentigo maligna	60	2.3
Spitzoid	58	2.2
Acral-lentiginous	48	1.8
Desmoplastic	11	0.4
Arising from blue naevus	1	0.04
Growth pattern		
Vertical	1,505	56.9
Radial	555	21.0
Unknown	587	22.1
Breslow thickness		
<0.75 mm	1,347	50.9
0.76–1.50 mm	514	19.4
1.51–3.99 mm	381	14.4
≥4 mm	253	9.6
Unknown	152	5.7
Ulceration		
Absent	2,026	76.5
Present	453	17.1
Unknown	168	6.4
Tumour regression		
Absent	1,160	43.8
Present	728	27.5
Unknown	759	28.7
TILs		
Present	1,789	67.6
Absent	504	19.0
Unknown	354	13.4
TNM stage		
I	1,827	69.02
II	379	14.32
III	261	9.86
IV	93	3.51
Unknown	87	3.29
Mitotic count (per mm^2^)		
Mean	2,6	
Median	1	
Min-Max	0-55	
Unknown	392	
Deceased		
No	2,285	86.32
In 1 year	98	3.70
In 2 years	88	3.32
In 3 years	90	3.40
In 4 years	86	3.25

**Figure 1:**
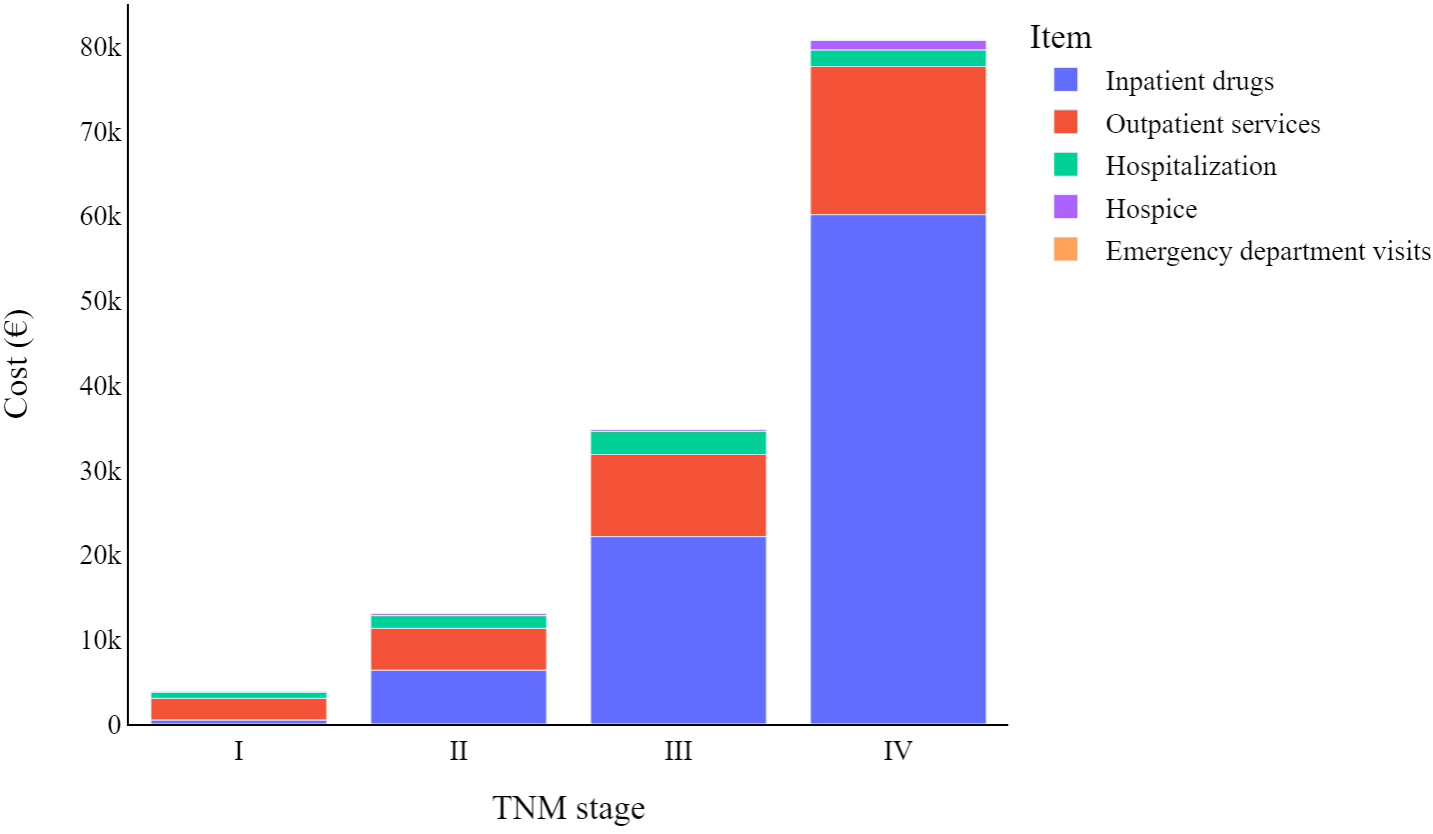
Total melanoma specific item cost (€) trends by TNM stage at diagnosis: from diagnosis to four years later.

**Figure 1** indicates how melanoma-related expenditures change based on tumour stage at diagnosis, with stage I CMM costing much less than stage IV. The increase in expenditure associated with inpatient drug use is the main driver of these different melanoma stage-related costs, followed by outpatient service costs. Lastly, hospice costs appear to primarily involve stage IV CMMs, while the impact of emergency department visits on total costs are negligible for all tumour stages.

**Figure 2:**
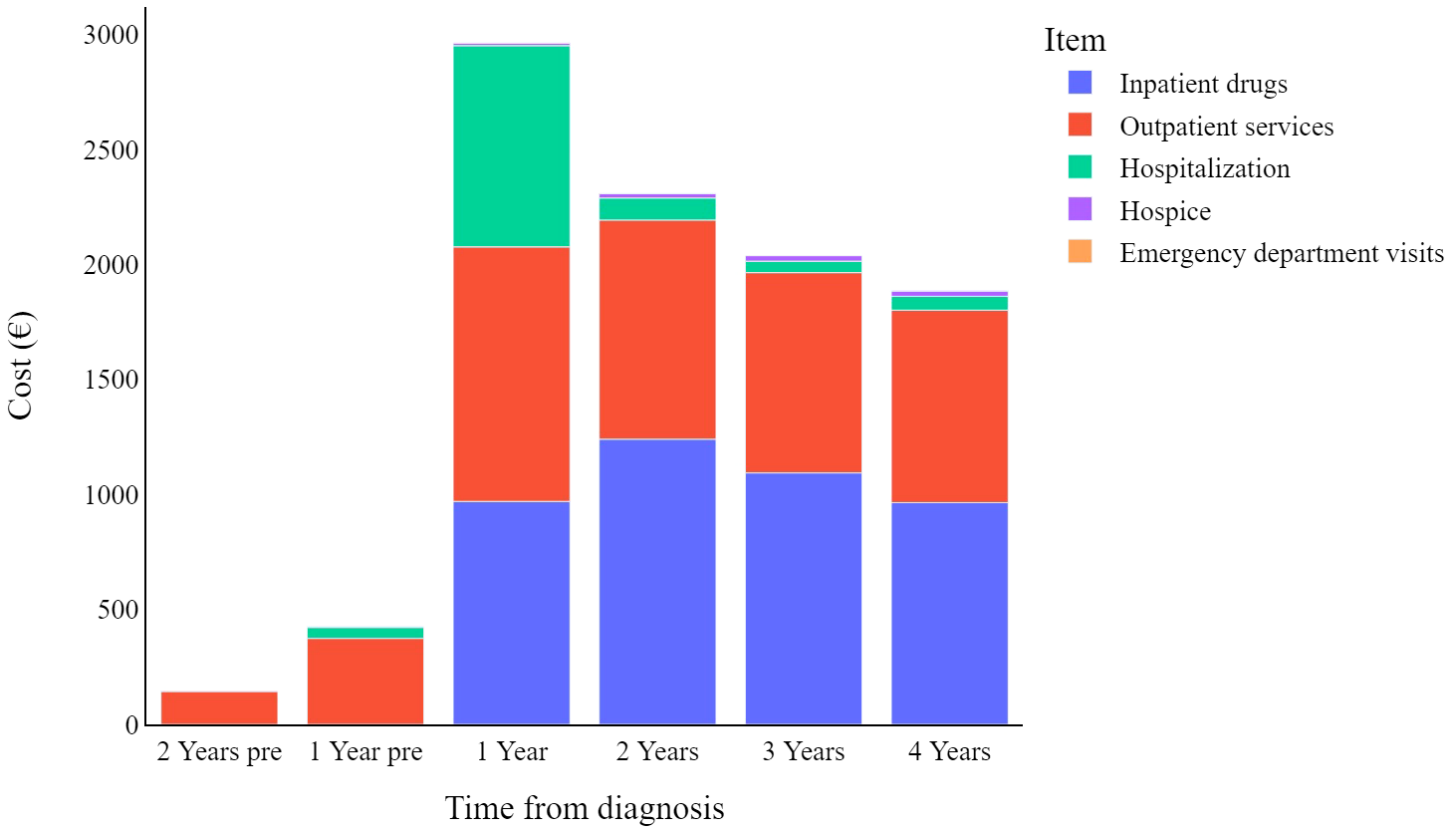
Melanoma specific item cost (€) trends by year pre- or post-diagnosis.

**Figure 2** displays the mean weighted direct melanoma-related costs per item per year, covering the period from 2 years prior to melanoma diagnosis until 4 years post-diagnosis. The mean value presented in **Table 2** represents the annual relative weight of each cost item within the total expenditure for CMM care, offering an immediate overview of the annual expenses across all categories. The data show that overall costs peak in the first year (€2,903, 95% C.I. [2,608-3,198]), and then decrease over time. Breaking it down further, in the first year hospitalization costs have a proportionally similar impact to that of inpatient drugs and outpatient services (€857, €950, and €1,084 respectively), while in subsequent years hospitalization costs are less impactful.

**Table 2:** Melanoma specific costs by expense item (€).

Time from diagnosis	Total		Inpatient drugs		Outpatient services		Hospitalization		Emergency department visits		Hospice care	
	Mean (95% C.I.)	Median [Min, Max]	Mean (95% C.I.)	Median [Min, Max]	Mean (95% C.I.)	Median [Min, Max]	Mean (95% C.I.)	Median [Min, Max]	Mean (95% C.I.)	Median [Min, Max]	Mean (95% C.I.)	Median [Min, Max]
2 Years pre	144 (129, 160)	20 [0, 8977]	–	–	144 (129, 160)	20 [0, 8977]	–	–	–	–	–	–
1 Year pre	425 (391, 459)	180 [0, 24637]	0 (0, 0)	0 [0, 173]	374 (342, 406)	173 [0, 24637]	48 (36, 59)	0 [0, 5878]	0 (0, 1)	0 [0, 617]	3 (3, 8)	0 [0, 7140]
1 Year	2903 (2608, 3198)	1271 [0, 105808]	950 (697, 1202)	0 [0, 91770]	1084 (1023, 1145)	584 [0, 20579]	857 (803, 911)	0 [0, 12224]	1 (0, 1)	0 [0, 409]	11 (0, 23)	0 [0, 12390]
2 Years	2260 (1923, 2597)	472 [0, 96235]	1214 (912, 1515)	0 [0, 90781]	933 (869, 996)	452 [0, 20302]	94 (73, 115)	0 [0, 9210]	1 (0, 2)	0 [0, 500]	19 (6, 32)	0 [0, 9030]
3 Years	2026 (1692, 2360)	405 [0, 89974]	1100 (814, 1385)	0 [0, 83177]	853 (781, 926)	398 [0, 25105]	49 (33, 64)	0 [0, 8023]	1 (0, 1)	0 [0, 410]	23 (8, 39)	0 [0, 11550]
4 Years	1861 (1557, 2164)	403 [0, 93497]	961 (697, 1226)	0 [0, 91368]	818 (754, 881)	396 [0, 18587]	58 (43, 74)	0 [0, 6291]	1 (0, 1)	0 [0, 668]	23 (8, 37)	0 [0, 8820]

**Figure 3:**
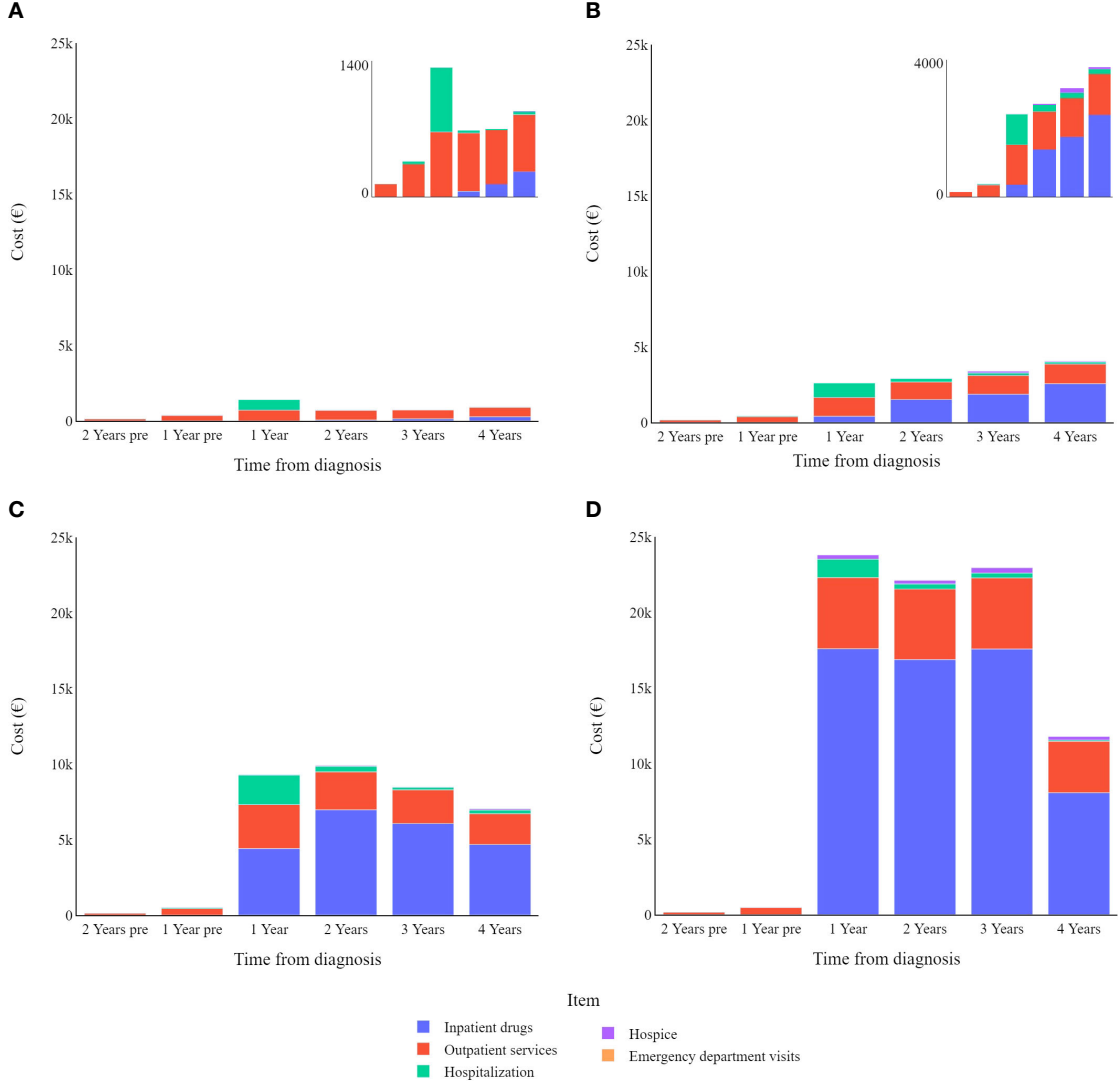
Melanoma specific item cost (€) trends by tumour stage (differentiated scales): **(A)** in stage I; **(B)** in stage II; **(C)** in stage III; **(D)** in stage IV.

Stratifying patients by disease stage at diagnosis (**Figure 3**) highlights the yearly relative weight of each cost-item in the overall expenditure for CMM care. Hospitalization contributes proportionally more to the total annual cost in the first year after diagnosis particularly for stage I CMM, while outpatient services have a proportionally similar impact on annual costs, and inpatient drugs show more variation in stage/year specific costs.

**Table 3** represents data on direct total costs of melanoma over a four-year period post-diagnosis, categorized by expense item and stratified by stage, age, sex, survival time, histological subtype, and overall costs.

**Table 3:** Direct total costs of melanoma (€) in the four years after diagnosis by expense item stratified by stage for age, sex, survival time, histological subtype and overall.

n				Total		Inpatient drugs		Hospitalization		Emergency department visits		Hospice care		Outpatient services	
				Mean (95% C.I.)	Median [IQR]	Mean (95% C.I.)	Median [IQR]	Mean (95% C.I.)	Median [IQR]	Mean (95% C.I.)	Median [IQR]	Mean (95% C.I.)	Median [IQR]	Mean (95% C.I.)	Median [IQR]
**All**		**All**	2647	9194 (7932, 10457)	2563 [4244]	4266 (3170, 5362)	0 [0]	1080 (974, 1187)	0 [1730]	3 (1, 5)	0 [0]	78 (22, 134)	0 [0]	3767 (3506, 4028)	1834 [2865]
		**Stage I**	1827	3807 (3309, 4305)	1911 [3143]	476 (129, 824)	0 [0]	770 (685, 856)	0 [1730]	0 (0, 1)	0 [0]	9 (0, 22)	0 [0]	2551 (2349, 2753)	1582 [1956]
		**Stage II**	379	13037 (9374, 16699)	3935 [6879]	6352 (3197, 9506)	0 [0]	1497 (1124, 1870)	0 [1730]	6 (0, 12)	0 [0]	242 (13, 471)	0 [0]	4941 (4192, 5689)	2706 [4344]
		**Stage III**	261	34759 (26083, 43434)	9666 [14635]	22119 (14270, 29968)	0 [3253]	2721 (2103, 3339)	734 [3861]	16 (0, 35)	0 [0]	214 (0, 500)	0 [0]	9689 (8228, 11151)	5975 [7844]
		**Stage IV**	93	80726 (52488, 108963)	16006 [87928]	60120 (34496, 85745)	0 [64905]	1959 (918, 3001)	0 [799]	49 (13, 86)	0 [0]	1119 (0, 2618)	0 [0]	17478 (12896, 22060)	9229 [17677]
**Age group**	**<64**	**All**	1569	8179 (6629, 9728)	2423 [3772]	3674 (2333, 5015)	0 [0]	1024 (898, 1149)	0 [1730]	3 (0, 5)	0 [0]	37 (0, 83)	0 [0]	3442 (3122, 3762)	1761 [2549]
		**Stage I**	1227	3581 (3030, 4131)	1892 [3017]	413 (74, 752)	0 [0]	788 (684, 891)	0 [1730]	0 (0, 0)	0 [0]	3 (0, 10)	0 [0]	2376 (2140, 2612)	1550 [1819]
		**Stage II**	126	13390 (6755, 20025)	4546 [6425]	6836 (1240, 12433)	0 [426]	1380 (840, 1919)	0 [1730]	10 (0, 26)	0 [0]	159 (0, 454)	0 [0]	5006 (3784, 6227)	3194 [3958]
		**Stage III**	134	37465 (25086, 49844)	11683 [14806]	24229 (12901, 35558)	0 [4193]	2749 (1927, 3571)	1336 [4012]	13 (0, 34)	0 [0]	158 (0, 384)	0 [0]	10315 (8269, 12362)	6436 [8406]
		**Stage IV**	34	86511 (36257, 136765)	19225 [108002]	65348 (20599, 110097)	108 [82537]	2263 (230, 4297)	0 [1342]	45 (0, 102)	0 [0]	861 (0, 2342)	0 [0]	17994 (10832, 25156)	10255 [16241]
	**≥65**	**All**	1078	10856 (8726, 12986)	2837 [5375]	5222 (3366, 7078)	0 [0]	1175 (988, 1363)	0 [1730]	4 (0, 8)	0 [0]	147 (31, 264)	0 [0]	4307 (3864, 4750)	1979 [3626]
		**Stage I**	600	4285 (3369, 5201)	1950 [3457]	605 (0, 1242)	0 [0]	735 (589, 880)	0 [1730]	0 (0, 1)	0 [0]	22 (0, 50)	0 [0]	2923 (2545, 3301)	1633 [2280]
		**Stage II**	253	12848 (8512, 17185)	3515 [6943]	6065 (2283, 9846)	0 [0]	1574 (1093, 2056)	0 [1730]	3 (0, 8)	0 [0]	291 (0, 590)	0 [0]	4915 (3970, 5861)	2464 [4715]
		**Stage III**	127	30877 (19125, 42630)	7747 [14176]	18994 (8550, 29437)	0 [1224]	2710 (1761, 3659)	286 [2449]	20 (0, 47)	0 [0]	267 (0, 664)	0 [0]	8887 (6911, 10863)	5337 [7218]
		**Stage IV**	59	75706 (41597, 109816)	16745 [58521]	55358 (23949, 86766)	74 [36417]	1724 (723, 2726)	0 [433]	56 (6, 105)	0 [0]	1270 (0, 2895)	0 [0]	17299 (10682, 23916)	8395 [17568]
**Sex**	**Female**	**All**	1243	7804 (6218, 9390)	2344 [3837]	3278 (1886, 4669)	0 [0]	1010 (866, 1155)	0 [1730]	2 (0, 5)	0 [0]	54 (0, 118)	0 [0]	3460 (3120, 3799)	1795 [2571]
		**Stage I**	899	3745 (3065, 4426)	1926 [3067]	457 (18, 897)	0 [0]	753 (634, 872)	0 [1730]	0 (0, 0)	0 [0]	1 (0, 4)	0 [0]	2534 (2256, 2812)	1611 [1921]
		**Stage II**	157	12411 (6479, 18343)	3894 [7510]	6307 (998, 11616)	0 [0]	1473 (903, 2043)	0 [1730]	2 (0, 4)	0 [0]	130 (0, 308)	0 [0]	4500 (3535, 5465)	2484 [4162]
		**Stage III**	98	33345 (20655, 46036)	9757 [17366]	20755 (9182, 32327)	0 [3164]	2769 (1747, 3790)	0 [4012]	20 (0, 53)	0 [0]	317 (0, 868)	0 [0]	9485 (7348, 11623)	5760 [10328]
		**Stage IV**	43	60735 (24283, 97186)	9580 [72244]	42780 (10238, 75321)	0 [41980]	2008 (553, 3463)	0 [1433]	47 (0, 99)	0 [0]	1189 (0, 3275)	0 [0]	14711 (8215, 21207)	6380 [16844]
	**Male**	**All**	1404	10443 (8522, 12363)	2833 [4688]	5153 (3495, 6811)	0 [0]	1143 (988, 1297)	0 [1730]	4 (1, 7)	0 [0]	101 (16, 186)	0 [0]	4042 (3653, 4431)	1899 [3174]
		**Stage I**	928	3866 (3173, 4559)	1905 [3230]	494 (29, 959)	0 [0]	788 (668, 907)	0 [1730]	0 (0, 1)	0 [0]	17 (0, 43)	0 [0]	2567 (2278, 2856)	1541 [1991]
		**Stage II**	222	13465 (8825, 18105)	3902 [6494]	6375 (2523, 10228)	0 [0]	1514 (1025, 2003)	0 [1730]	8 (0, 19)	0 [0]	320 (0, 670)	0 [0]	5247 (4172, 6322)	2769 [4349]
		**Stage III**	163	35619 (23870, 47368)	9441 [12952]	22950 (12366, 33534)	0 [3273]	2693 (1923, 3463)	865 [3661]	14 (0, 31)	0 [0]	152 (0, 405)	0 [0]	9810 (7842, 11779)	6062 [7171]
		**Stage IV**	50	97056 (53231, 140882)	22026 [105429]	74398 (34079, 114717)	3440 [84437]	1913 (482, 3344)	0 [0]	51 (0, 105)	0 [0]	1026 (0, 2514)	0 [0]	19668 (13230, 26107)	13616 [18229]

**Figure 4:**
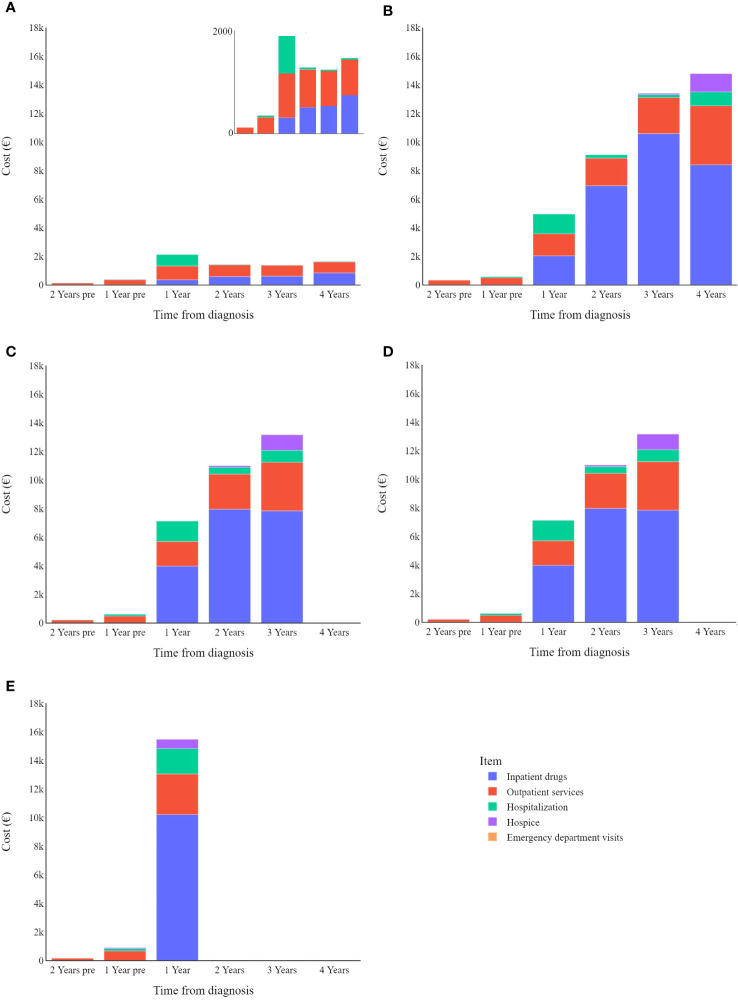
Melanoma specific item cost (€) trends by survival: **(A)** in survived; **(B)** in deceased in 4 years; **(C)** in deceased in 3 years; **(D)** in deceased in 2 years; **(E)** in deceased in 1 years.

**Figure 4** shows that patients that survived less than four years have an annual expenditure mean of €11,827, compared to a mean of €1,648 for those who survived longer than the observation period.

Lastly, multivariable regression analysis (**Table 4**) shows that TNM stage at diagnosis and growth pattern were independently associated with cost changes, both for overall costs as well as cost per year for each of the four years following diagnosis. On the other hand, mitotic count, tumour regression, and primary tumour location were significantly associated with the year-specific cost but not when considering specific years. Lastly, the time to death in the index year resulted in statistically significant changes in costs.

**Table 4:** Log-linear regression analysis of the association between person-years melanoma-specific direct costs (at specific times from diagnosis as well as covering the whole observed period) and age, sex, time to death, TNM stage at diagnosis, mitotic count, TILs, tumour regression, ulceration, growth phase, tumour location, and histology subtype.

	1 year		2 years		3 years		4 years		Total	
	Exp (coeff)	p-value	Exp (coeff)	p-value	Exp (coeff)	p-value	Exp (coeff)	p-value	Exp (coeff)	p-value
Age group (reference <64)										
≥65	0.95	0.481	1.16	0.133	1.08	0.453	1.03	0.780	0.98	0.724
Sex (reference Female)										
Male	0.95	0.469	0.92	0.393	1.06	0.559	0.87	0.161	1.03	0.699
Time to death (reference No)										
1 year	0.80	0.331	–	**-**	–	**-**	–	**-**	**-**	**-**
2 years	0.73	0.175	1.72	0.083	–	**-**	–	**-**	**-**	**-**
3 years	0.80	0.284	1.31	0.325	1.71	0.053	–	**-**	**-**	**-**
4 years	1.02	0.922	1.73	**0.037**	4.01	**<0.001**	2.15	**0.006**	**-**	**-**
TNM stage (reference I)										
II	1.36	**0.028**	1.41	0.068	1.70	**0.006**	1.38	0.109	1.49	**0.002**
III	4.77	**<0.001**	4.61	**<0.001**	3.13	**<0.001**	2.74	**<0.001**	4.70	**<0.001**
IV	8.76	**<0.001**	8.95	**<0.001**	5.42	**<0.001**	4.06	**0.002**	5.64	**<0.001**
Mitotic count (per mm^2^)										
	1.02	**0.035**	1.01	0.208	1.00	0.818	1.01	0.356	1.01	0.114
TILs (reference Absent)										
Present	0.95	0.562	0.84	0.121	1.01	0.924	0.87	0.233	0.87	0.072
Tumour regression (reference Absent)										
Present	0.91	0.189	0.96	0.677	0.77	**0.011**	0.98	0.831	0.89	0.083
Ulceration (reference Absent)										
Present	0.95	0.689	1.01	0.971	0.904	0.554	0.97	0.863	1.14	0.233
Growth pattern (reference Radial)										
Vertical	1.39	**<0.001**	1.35	**0.006**	1.22	**0.077**	1.27	**0.038**	1.39	**<0.001**
Primary site (reference lower limb)										
Upper limb	1.19	0.137	1.53	**0.007**	1.00	0.990	1.21	0.270	1.10	0.392
Head	1.15	0.315	1.28	0.183	0.86	0.408	0.90	0.579	1.08	0.538
Hands/feet	0.95	0.758	1.49	0.084	1.19	0.466	1.29	0.320	0.88	0.441
Trunk	0.96	0.582	1.45	**0.003**	1.00	0.988	1.07	0.606	0.99	0.882
M. Histology Subtype (reference nodular)										
Non-nodular	0.97	0.815	1.03	0.848	1.00	0.985	0.97	0.848	0.96	0.744

p-values below the significance level (<0.05) are formatted in boldface type.

## 4 Discussion

The current study indicated that cost items associated with cutaneous malignant melanoma can be better understood by grouping patients according to their tumour characteristics. Specific cost items contribute differently to the total cost of care for the patient over time and are also related to the clinical and anatomopathological characteristics of the tumour.

### 4.1 Yearly cost trends after diagnosis

Overall, analysing all data together, CMM patients tend to have higher costs in the first year after diagnosis, which then gradually decrease in subsequent years. As shown in the stage-specific sub analysis, this is only true when considering all CMM patients together, regardless of the stage of the disease at diagnosis (**Figure 3**).

In a previous publication analysing direct health care costs in patients with cutaneous malignant melanoma, Lyth and colleagues also reported a similar decrease in mean costs over time, both overall and depending on the clinical stage at diagnosis (15). Our data further reveal specific trends, by grouping costs into cost items (e.g., hospitalization, outpatient services, inpatient drugs, etc.). It should be noted here that under the Italian NHS, the reported costs of care are equal to the sum of the individual fees for each medical procedure provided to the patient, which are fixed amounts defined *a priori* by regional regulations. Thus, it can be observed that hospital-related costs peak in the first year after diagnosis and then gradually decrease over the years, suggesting that hospitalization is mainly considered at the beginning of treatment. In contrast, hospice-related spending shows the opposite trend, as patients with CMM could benefit from such care towards the end of their lives. The reason why hospital drug costs peak two years after diagnosis and then decline in subsequent years deserves to be investigated in future studies, as there is no obvious explanation either from the scientific literature or from analysis of the clinical care pathway established by regional guidelines (27). Finally, spending associated with outpatient services and emergency department visits does not seem to follow any obvious pattern that can be analysed.

### 4.2 Overall costs based on stage

As is known, advanced tumour stages are associated with higher costs of care (14, 15, 28, 29). Indeed, patients in our cohort that had stage IV CMMs were associated with costs that were more than 20 times higher than those with stage I (€80,726 vs. €3,807). Two other studies also reported melanoma-related costs of care according to tumour stage and time at diagnosis – these 2016 studies were carried out by Serra-Arbeloa et al. (14) and Lyth et al. (15). They drew data from the official Spanish national health system reports and the Swedish cancer registry (respectively). A comparison of first-year melanoma treatment costs shows that for stage I melanoma patients, we in Italy show the lowest first-year costs (€1,401), compared to the Spanish (€3,103) and the Swedes (€2,670). For stage IV patients, the Swedes reported the lowest expenditure (€29,291), followed by our present study (€30,762), with the Spanish reporting significantly higher expenditure than both (€88,268).

### 4.3 Cost items by stage at diagnosis

In most cases, the main cost driver is inpatient drugs, however this is not the case for stage I CMMs, which instead show higher costs related to outpatient services. The relative impact of hospitalization is greater for lower cancer stages because other cost items are relatively limited in these early stages of the disease. Even though it should be noted that hospitalizations are (in absolute value) costlier for more advanced melanomas, this drops slightly for stage IV melanomas.

As stated by Cullison C.S. and Bordeaux J.S. in a letter to the editor, excision of primary cutaneous melanoma is a low-risk operation usually associated with few complications, therefore hospitalization is not an expected consequence (30). While patients with stage I CMM often recover after the first tumour removal, more advanced melanoma patients face higher recurrence rates, which may influence the course of their disease in subsequent years and thus also increase costs (8, 31–33). Indeed, Leenaman and colleagues reported a 47% recurrence rate for stage III melanoma patients, compared to 29% for stage II and 8% for stage I (33). In stage IV advanced metastatic cutaneous malignant melanomas, surgical or other ablative treatments are limited to palliative care or as a complementary treatment in a combined approach to oligometastases (20, 23, 34–38).

Other costs four years from diagnosis differ widely among melanoma stages. Data highlight how the use of inpatient drugs heavily influences the cost of care for CMM, moving from an average cost of just over €6,000 for stage II melanomas to nearly €50,000 for stage IV melanomas. It should be noted that emergency department visits are always a minor factor in the total cost associated with CMM care, always accounting for less than 1% of the total expenditure.

### 4.4 Costs near the end of life

Overall, data show that death increases healthcare costs associated with CMM, where patients who survive beyond the observation period show substantially lower annual expenditure than patients who succumb to their disease. Specifically, patients who died during the observation period had the highest overall costs in their last year of life. In a recent literature review, Pisu and colleagues (39) summarized healthcare expenditure related to cancer care for multiple tumour types, stating that the initial and end-of-life phases are associated with the highest expenditure (both direct and out-of-pocket costs), while costs over the middle phases are generally lower. In an older systematic review covering articles from 1990-2011, Guy and colleagues (28) analysed the cost of melanoma care, highlighting that healthcare costs tend to be highest in the terminal phase and lowest in the interim phase (largely according to two studies conducted in the US). Consistently our present study indicates that end-of-life costs are highest and increased by hospice care (peaking in the last year of the patients’ lives) as well as hospitalization-related costs (which are higher than those seen in intermediate phases).

### 4.5 Other anatomo-pathological factors

Other factors can also significantly influence costs associated with CMM care (both overall and per year in the four years following diagnosis), such as tumour growth patterns. As hypothesized several decades ago, tumour progression in melanomas goes through successive stages of growth, from radial to vertical to metastatic disease (40–42). Invasion of the lower layers of the dermis has been associated with increased tumour aggressiveness, also linked by some authors to the expression of specific biological markers by the tumour (42). Consequently, it can be argued that the higher costs of vertically growing CMMs may be a consequence of the higher intensity of care required due to the more aggressive behaviour of these tumours (14, 43). However, this direct association between tumour growth pattern, treatment intensity and overall cost of care needs specific investigation to test its validity.

### 4.6 Limitations and strengths

The present study comes with the typical limitations and strengths seen in cohort studies that are based on regional, linked administrative databases. Even if a defined clinical pathway is common between different healthcare facilities in a region, the simple fact of including subjects that were treated in different hospitals could have distorted the results, not to mention the effect of dataset inaccuracies or missing values. Furthermore, due to the structure of these databases, our study could not explore the indirect costs of cutaneous malignant melanoma, thus limiting the goal of the research. Further studies are also needed to analyse cost differences after recent changes to the CMM treatment paradigm and the introduction of immunotherapies.

In contrast, the main strength of this study lies in its population-based design, which enables it to provide information on costs retrieved from real-world clinical practice, while minimizing the risk of selection bias that can occur with independently collected data.

## 5 Conclusion

Indeed, policy makers could greatly benefit from the analysis of CMM-related expenditure over time. In fact, a rational and judicious allocation of healthcare expenditures should account for the direct costs incurred in patient care pathways accordantly with results evidenced by health economic research. Unfortunately, researches based on expenditure determinants are still scarce. With better knowledge, they would be able to consider specific costs (i.e., overall, stage specific, and item specific costs, plus how they change over time) when allocating resources for CMM patients, in order to provide the best management of the disease and the best quality of care.

## Data availability statement

The data supporting this study’s findings are held by the Veneto Epidemiological Registry and were used under license for this work, but they are not available to the general public. The original contributions presented in the study are included in the article/supplementary material. Further inquiries can be directed to the corresponding author and subject to authorization from the Veneto Epidemiological Registry (Veneto Regional Authority).

## Ethics statement

The studies involving humans were approved by Veneto Oncological Institute’s Ethics Committee. The studies were conducted in accordance with the local legislation and institutional requirements. The participants provided their written informed consent to participate in this study.

## Author contributions

Conceptualization, AB; methodology, CC; software, CC; formal analysis, CC; investigation, CC; data curation, CC; visualization, CC; writing - original draft preparation, AB and AM; writing - review and editing, AB, AM and CC; supervision, AB; project administration, AB; All authors contributed to the article and approved the submitted version.
